# Common Musculoskeletal Disorders in the Elderly: The Star Triad

**DOI:** 10.3390/jcm9041216

**Published:** 2020-04-23

**Authors:** Marco Alessandro Minetto, Alessandro Giannini, Rebecca McConnell, Chiara Busso, Guglielmo Torre, Giuseppe Massazza

**Affiliations:** 1 Division of Physical Medicine and Rehabilitation, Department of Surgical Sciences, University of Turin, 10126 Turin, Italy; marco.minetto@unito.it (M.A.M.); alessandro.giannini@unito.it (A.G.); 2rebeccamcconnell@gmail.com (R.M.); chiara.busso@unito.it (C.B.); giuseppe.massazza@unito.it (G.M.); 2 Department of Orthopaedic And Trauma Surgery, Campus Bio-Medico University of Rome, 00128 Rome, Italy

**Keywords:** aging, osteoarthritis, sarcopenia, tendinopathies

## Abstract

Musculoskeletal disorders are debilitating conditions that significantly impair the state of health, especially in elderly subjects. A pathological triad of inter-related disorders that are highly prevalent in the elderly consists of the following main “components”: sarcopenia, tendinopathies, and arthritis. The aim of this review is to critically appraise the literature relative to the different disorders of this triad, in order to highlight the pathophysiological common denominator and propose strategies for personalized clinical management of patients presenting with this combination of musculoskeletal disorders. Their pathophysiological common denominator is represented by progressive loss of (focal or generalized) neuromuscular performance with a risk of adverse outcomes such as pain, mobility disorders, increased risk of falls and fractures, and impaired ability or disability to perform activities of daily living. The precise management of these disorders requires not only the use of available tools and recently proposed operational definitions, but also the development of new tools and approaches for prediction, diagnosis, monitoring, and prognosis of the three disorders and their combination.

## 1. Introduction

Musculoskeletal disorders are debilitating conditions that significantly impair the state of health, especially in elderly subjects, since they are associated with pain, mobility disorders, increased risk of falls and fractures, and impaired ability or disability to perform activities of daily living. A pathological triad of inter-related disorders that are highly prevalent in elderly subjects consists of the following main “components”: sarcopenia, tendinopathies, and arthritis (the acronym “STAR” will be henceforth adopted).

Interdependency within the different components of the triad fuels an accelerating disease progression that culminates in motor impairment, reduced quality of life, and increased risks of disability, morbidity, and mortality. Clinical and experimental findings show the interdependency within the three disorders. In fact, quadriceps weakness increases the risk of knee and hip osteoarthritis and also increases disease- and treatment-related complications [1,2]. Similarly, poor abductor hip function and low physical performance are known risk factors for gluteal tendinopathy [3,4]. Recent studies also showed that motor impairments (that are common in patients with both lower limb tendinopathies and hip or knee osteoarthritis) may predispose to sarcopenia and contribute to its progression [5]. Furthermore, age-related comorbidities, such as chronic obstructive pulmonary disease and congestive heart failure, can limit mobility resulting in decreased muscle and tendon function, thus propagating changes already occurring in the musculoskeletal system [6]. Although common pathways have been implicated in the pathogenesis of the different components of the triad [6], these diseases are rarely evaluated in a comprehensive manner [6] and, to the best of our knowledge, no previous study has investigated the frequency of comorbidity of sarcopenia, tendinopathies, and arthritis.

An example of a clinical case that is commonly seen in daily practice, highlighting the possible interdependency of the STAR triad’s musculoskeletal disorders is as follows. A 70-year-old female presents to her general practitioner with complaints of difficulty in moving and pain. She reports that it is becoming difficult to rise from a chair and she sometimes loses her balance when walking on uneven surfaces. Reaching cups on the high kitchen shelves causes shoulder pain, and her knees, which used to ache only in the morning, now hurt all the time. Though she had multiple minor musculoskeletal injuries in her youth and worked in a factory for twenty years, she had only a few medical concerns during adulthood. After she retired, however, her activity level declined, and controlling her weight gain and diabetes has been difficult. Her doctor is concerned that multiple musculoskeletal disorders are affecting her independence and quality of life.

The aim of this narrative review is to critically appraise the literature relative to the different disorders of this “STAR” triad, in order to highlight the pathophysiological common denominators and propose strategies for personalized clinical management of patients presenting with this combination of musculoskeletal disorders. Literature research was performed, including all relevant studies up to January 2020 by searching the Medline/PubMed database and Web of Science using the following search terms: arthritis, muscle weakness, musculoskeletal pain, osteoarthritis, physical frailty, sarcopenia, tendinopathy, tendon injury, and healing.

## 2. Sarcopenia

### 2.1. Definition

Sarcopenia is the loss of skeletal muscle mass and function that occurs during the aging process (primary sarcopenia) or due to the presence of an underlying disease or medication (secondary sarcopenia). While secondary sarcopenia relies on the diagnosis and treatment for the underlying causation, primary sarcopenia has been more challenging to characterize. The European Working Group on Sarcopenia in Older People described sarcopenia as “a syndrome characterized by progressive and generalized loss of skeletal muscle mass and strength with a risk of adverse outcomes such as physical disability, poor quality of life, and death” [7].

### 2.2. Epidemiology

Epidemiological studies showed that from the second to the eighth decade of life, whole body and appendicular lean mass decline by about 20% in men and 30% in women [8,9]. Sarcopenia, therefore, is prevalent in older adults, especially women, and presents substantial variations depending on age and geographic area. A 2014 systematic review reported the following sarcopenia prevalence data within different healthcare settings: 1%–29% in the community, 10% in acute hospital care, and 14%–33% in long-term care setting [10].

### 2.3. Pathophysiology

From a pathophysiologic perspective, sarcopenia can be considered as an organ failure: Correa-de-Araujo and Hadley [11] proposed the concept of “skeletal muscle function deficit”, while Marzetti et al. [12] proposed the pathophysiologic construct of “muscle insufficiency”.

This organ failure can develop chronically (more often) or acutely (e.g., during immobilization) and results from a combination of neural and muscular adaptations [13,14]. The former adaptations consist of neuropathic processes leading to motor unit denervation and preferential loss of fast motor units. The latter adaptations consist of a loss of muscle mass that is due to a decrease in muscle fiber number (hypoplasia) and size (atrophy), which particularly affects type 2 fibers (that are more vulnerable to atrophy than type 1 fibers). Given the abovementioned preferential loss of fast motor units (containing fast type 2 fibers), the muscular adaptations could also imply a fast-to-slow transition of fiber types (i.e., a change of the myosin heavy chain (MHC) isoform expression toward the slower phenotype) [13]. However, MHC expression is also affected by the level of neuromuscular activity: disuse favors the expression of fast MHC isoforms, while physical activity leads to either a fast-to-slow (glycolytic-to-oxidative) phenotype transition or a shift toward the slower population of fast fibers (i.e., a bidirectional transformation from MHC-1 and MHC-2X isoforms toward MHC-2A isoform) [13,14,15]. This variability in muscle adaptations to physical activity is a function of the pre-training MHC phenotype, training history, and training type. Hence, an MHC phenotype characteristic of aging does not exist since it is the result of the complex interaction between age-related neurodegenerative changes and physical activity status, which varies across different individuals.

### 2.4. Clinical Presentation

The European Working Group on Sarcopenia in Older People proposed in 2010 an operational definition of sarcopenia based on the co-occurrence of low muscle mass and low muscle function (strength or performance) [7]. This operational definition has recently been updated as follows: (1) low muscle strength is identified as a key characteristic and a primary indicator of probable sarcopenia; (2) sarcopenia diagnosis is confirmed by the presence of low muscle quantity or quality; and (3) detection of low physical performance predicts adverse outcomes, so such measures are thus used to identify the severity of sarcopenia. Therefore, when low muscle strength, low muscle quantity/quality, and low physical performance are all detected, sarcopenia is considered to be severe [16].

Other consensus groups (International Working Group on Sarcopenia (IWGS), Society of Sarcopenia, Cachexia and Wasting Disorders (SSCWD), Foundation for the National Institutes of Health Sarcopenia Project, and Asian Working Group on Sarcopenia) agree that the diagnosis of sarcopenia should incorporate a muscle mass evaluation with the assessment of strength and/or physical performance [17,18,19,20].

Therefore, the reduction in muscle size and strength and neuromuscular performance impairment must be considered as core clinical presentations of sarcopenia: a critical mass of these phenotypic components identifies and evaluates the severity of the syndrome. This operationalization of the sarcopenic syndrome resembles the definition of the frailty phenotype previously proposed by Fried et al. [21] that is based on the co-occurrence of (three or more of) the following criteria: unintentional weight loss, self-reported exhaustion, muscle weakness (low grip strength), slow walking speed, and low physical activity. Most of these criteria are the same required for the diagnosis of sarcopenia. Consistently, sarcopenia must be considered as a key component of frailty [22].

In addition to the formal clinical presentation framework, a recent stated-preference study performed in community dwelling, elderly, sarcopenic patients provided insights on the clinical features of sarcopenia that are relevant from the patients’ perspective, including mobility impairment, inability to manage domestic activities, increased risk of falls, fatigue, and reduced quality of life [23].

Therefore, treatment interventions for sarcopenic patients should be performed to address not only the core phenotypic components of the syndrome (the impairments of muscle mass and function), but also the associated negative health outcomes.

### 2.5. Management

Understanding the pathophysiology and core clinical characteristics of sarcopenia is key to developing effective interventions, and translational research in this area is rapidly increasing [24]. Current management strategies include non-pharmacological and pharmacological approaches. Physical exercise, alone or in combination with nutritional interventions, is the non-pharmacological approach currently recommended as the primary treatment of sarcopenia [25]. In fact, physical exercise, most notably high-intensity resistance training, improves the strength and mass of skeletal muscles and counteracts the age-related decline in muscle size and function [24,25,26]. It is, however, worth noting that the feasibility, sustainability, and safety of resistance training in individuals with sarcopenia deserve further investigation [26], especially because high-intensity resistance exercises increase the risk of muscle and tendon injuries and can also produce post-exercise muscle soreness and persistent fatigue. These symptoms (that also represent the core clinical characteristics of overtraining syndrome) [27] should be avoided in elderly subjects as they can be associated with reduced motor performance, mood changes, and poor quality of life. As older individuals seem to prefer easy and accessible training regimens that are easy to perform in any setting, body weight-based exercise programs for strength training may be preferable to programs involving gym equipment [28]. Current recommendations for physical exercise prescription in sarcopenic and frail older people include a balanced program of both endurance and strength exercises, performed on a regular schedule [29,30].

The effects of exercise may be enhanced by a wide variety of other treatments, including patient education (patients must be instructed to progressively increase training load and to train at a high intensity) [26] and nutritional supplements providing an adequate intake of protein, vitamin D, antioxidant nutrients, and long-chain polyunsaturated fatty acids [31].

Furthermore, evidence for the benefits of different physical therapies in improving muscle strength and mass individually is compelling, and evidence for their benefit in sarcopenia is growing. For example, neuromuscular electrical stimulation (i.e., the application of high-intensity and intermittent electrical stimuli to the skin above the muscles, with the main objective to generate involuntary muscle contractions, most often in isometric tetanic conditions) [32], whole body vibration (i.e., the use of a vertical or rotary oscillating platform as an exercise stimulus, while the individual engages in sustained static positioning or dynamic movements) [33], and focal muscle vibration (i.e., the application of pneumatic stimuli to the skin above the muscles, with the main objective to stimulate cutaneous and subcutaneous receptors without triggering visible muscle contractions) [34] proved to be effective in sarcopenic patients. However, the heterogeneity of the data regarding the adopted stimulation paradigms and training protocols does not yet allow firm conclusions to guide clinical recommendations for physical therapy prescription in sarcopenic patients.

Regarding pharmacological approaches, it is worth highlighting that no drugs have been approved yet for the treatment of sarcopenia. A recent umbrella review of systematic reviews and meta-analyses reported that only vitamin D, especially in older women, and testosterone in older men with low testosterone levels and muscle weakness can be justified in daily clinical practice to improve muscle mass and function in sarcopenic patients [35].

## 3. Tendinopathies

### 3.1. Definition

Tendinopathy is a musculoskeletal disorder characterized by tendon pain during an activity, tendon swelling and localized tenderness upon palpation, and loss of function [36]. Different medical terms are frequently used to define this disorder [37] and this ambiguity reflects the controversy that surrounds pathogenesis of tendinopathy. The traditional assumption was that tendon injury may result from repetitive mechanical loads and subsequent inflammatory responses (hence the term “tendinitis”) [38]. However, histopathological findings were unable to consistently find “classical” inflammatory cells. Instead, light microscopy investigation of samples of pathological tendons showed collagen degeneration, increased mucoid ground substance, and increased tenocytes with myofibroblastic differentiation (tendon repair cells) [39]. For this reason, “tendinosis” became the preferred term over “tendinitis” to avoid the implication of inflammation. Modern molecular techniques, however, have clearly shown the increased presence of macrophages and mast cells in tendinopathic tissues [40]. Therefore, chronic, low-grade inflammation may still be part of the pathogenesis [41,42]. For all the reasons mentioned above, tendon experts recommend the use of “tendinopathy”, a term that does not imply the presence of a particular pathological or biochemical process [36,43,44].

### 3.2. Epidemiology

The most common tendinopathies in the elderly involve two tendons (i.e., subscapularis and supraspinatus) of the rotator cuff of the shoulder [45,46] and the gluteal tendons of the hip [47]. Rotator cuff tendinopathy prevalence in the elderly population ranges from 5% to 7% [45,46], and a recent study investigating tendinopathies in a Dutch general practice found gluteal tendinopathy to have the highest prevalence (4.22 per 1000 person years) and incidence (3.29 per 1000 person years) of all lower limb tendinopathies [48]. Therefore, the following sections will focus on the pathophysiology, clinical characteristics, and management strategies for degenerative rotator cuff tendinopathy and gluteal tendinopathy (also known as “greater trochanteric pain syndrome”).

### 3.3. Pathophysiology

Despite the progress achieved over the last decades, there is still great uncertainty over the factors causing this pathology and controlling its progression. A widely accepted concept for explaining the origin and development of tendinopathy is the generation of excessive tensile loads within the tendon over time. If a rapid increase in the magnitude, duration, or frequency of loading occurs, tensile strength might be exceeded locally causing a micro-injury. The mechanisms underlying the progression of this micro-injury are currently not established, but it has been hypothesized that repetitive overloading of the tendon may overwhelm the tissue’s healing capacity causing a more severe injury [43,44]. This concept identifies excessive tensile load to be the key factor in tendinopathy origin and development. However, a number of other findings indicated that there is more into the etiology and pathophysiology of tendinopathy than just excessive tensile load. For example, it has been shown in recent in vivo studies that human tendons may undergo non-uniform displacements during passive or active force application, with deeper tissue layers deforming more than superficial ones [49]. Therefore, not only excessive tensile stress, but also excessive compressive stress as well as “stress-shielding” (i.e., the lack of adequate local stress producing understimulation of the tendon cells), could lead to tensile weakening and degeneration over time [50,51]. For greater trochanteric pain syndrome, the role of compression seems to be clear: the gluteus medius and minimus tendons compress against the greater trochanter in positions of hip adduction [52]. Similarly, the deep fibers of the supraspinatus tendon are exposed to high compressive loads against the humeral insertion [53]. High levels of tendon compression and the associated tissue hypoxia stimulate the fibrocartilaginous metaplasia of tenocytes that differentiate into hypertrophic chondrocyte-like cells to produce calcium deposits (predominantly hydroxyapatite) [54,55]. The clinical sequela is a condition known as calcific tendinopathy [56]. The negative effects of the excess or lack of adequate local stress on the tendon can also be amplified by systemic factors such as metabolic alterations related to aging (i.e., obesity and type 2 diabetes) [57]. Therefore, age-related tendinopathies could be viewed as the result of a degenerative process underlain by local and systemic factors. However, neither the “failed healing model” nor the “degenerative model” of tendinopathy fully explains the heterogeneity of its presentation. The “continuum model” of tendon pathology was proposed in 2009 and revisited in 2016 by Cook et al. [58,59] to overcome the limitations of previous models. This model assumes that tendons may have discrete regions that are in different stages (normal, reactive, or degenerative) at one time. This model also assumes that regardless of the initiating event (overstimulation or understimulation of resident tenocytes, collagen disruption or micro-injury, or inflammation), tendon pathology is characterized by a significant cell response to injury. Therefore, a tendon cell-based response could occur in a structurally normal (to conventional imaging modalities at least) tendon portion that may drift in and out of a reactive response. Although the exact mechanisms responsible for tendon pain remain to be clarified [60], the “continuum model” also suggests that paracrine signaling by tenocytes could represent one of the drivers of nociception. In fact, cytokines released by tenocytes (and/or infiltrating immune cells) could sensitize peripheral mechanoreceptors near or in the paratenon (that could also be irritated by the increased tendon size) as well as nociceptor terminals, ultimately resulting in the stimulation of the peripheral nerve. However, mechanisms for tendon pain should extend beyond local tissue changes and include increased axonal sprouting from repeated injury as well as peripheral and central mechanisms of nociception modulation [60].

### 3.4. Clinical Presentation

Core clinical characteristics of rotator cuff degenerative tendinopathy include old age, focal night pain, weakness of shoulder muscles, and movement restriction [61,62]. Shoulder pain generally radiates to the deltoid and the middle part of the upper arm. Weakness, movement restriction, or both, especially in active external rotation, are found in half of radiologically confirmed rotator cuff tears [62]. Movement testing helps to distinguish rotator cuff degenerative tendinopathy from frozen shoulder (adhesive capsulitis). Frozen shoulder is an idiopathic and self-limiting condition in which movement of the shoulder becomes restricted in both active and passive external rotation [63].

Calcific tendinopathy is another important, self-limiting differential diagnosis for patients presenting with shoulder pain, especially in middle-aged patients. It is a condition in which calcium deposits develop in the supraspinatus (80% of cases), infraspinatus (15% of cases), and subscapularis (5% of cases) tendons [55,56]. Calcific tendinopathy can be diagnosed through imaging of calcium deposits and may respond differently to treatments compared to degenerative tendinopathies [55,56].

Like shoulder tendinopathy, low-grade pain (with or without movement restriction) is characteristic of gluteal tendinopathy. This pain presents in the lateral hip and is aggravated with activities (such as walking and other weight-bearing activities) and side-lying on the affected side [52]. Further core clinical characteristics of gluteal tendinopathy include old age, female gender, comorbidities such as pain generated from the back and hip joint, overweight/obesity, poor abductor hip function, altered gait parameters, and psychological distress [3,4].

### 3.5. Management

The best approaches for clinical management of rotator cuff and gluteal tendinopathies have yet to be elucidated. Careful analysis of the medical history and symptoms must guide clinical decision making. Studied interventions include exercise, interventional approaches (i.e., corticosteroid or platelet-rich plasma injections), physical therapy modalities (radial pressure wave treatment and focused shock wave therapy), and topical glyceryl nitrate application.

Positioning and exercise therapy seem to be effective in both rotator cuff tendinopathy and greater trochanteric pain syndrome; however, it is unclear what protocol is the best. Overuse may be managed by reducing loads and improving biomechanics. Reducing compression must be evaluated carefully in static and dynamic positioning. In greater trochanteric pain syndrome, it is probably useful to advise patients to avoid hip-adducted positions, such as standing with “hanging on one hip” or standing with crossed legs. In the same way, patients with rotator cuff tendinopathy should avoid sustained work positions where the shoulder is unsupported in abduction. Exercises commonly start with isometric contractions because they are easy to perform, well-tolerated, and may have analgesic benefits [64]. Once pain is more tolerable, then a restorative exercise program with an early and gradually progressive tensile loading may improve the tendon’s architecture and function. Increased loading improves the load-bearing capacity, and low-velocity, high-tensile load exercises benefit the tendon structure [65]. Eccentric exercises provide additional strain that transmit higher forces through the joint [66]. As the symptoms continue to improve, more functional exercises, such as jumping, running, and throwing, can be added progressively. More detailed exercise programs for both shoulder tendinopathies and greater trochanteric pain syndrome can be found in the literature [67,68].

For both gluteal tendinopathy and degenerative rotator cuff tendinopathy, peritendinous corticosteroid injection provides moderate pain relief, but only for a short time (less than 4 weeks) [69,70]. Recent preliminary evidence suggests that platelet-rich plasma injections may also clinically benefit patients with gluteal tendinopathy [71,72,73].

However, a physiotherapy-led education and exercise program performs better than corticosteroid injection in the long-term follow-up of gluteal tendinopathy patients and should be considered as first-line treatment [67]. Similar to the previous findings, Korakakis et al. [74] observed that corticosteroid injection is superior to radial pressure wave treatment in the short-term (1 month), but that physical therapy is superior to corticosteroid injection at mid-term (4 months) and long-term (>12 months) follow-up in gluteal tendinopathy patients. Moreover, focused shock wave therapy was effective in reducing lateral hip pain, both in the short-term (2 months) and mid-term (6 months) follow-up [75]. Given that the gluteal tendon insertion on the greater trochanter has variable depth to the skin surface, especially in female patients presenting a gynoid fat distribution [4,52], it is generally assumed that focused shock wave therapy is more effective compared with radial pressure wave treatment [76]. However, no previous study has compared these two modalities in the management of gluteal tendinopathy.

Regarding rotator cuff tendinopathy, there is consistent evidence that both focused shock wave therapy and radial pressure wave treatment reduce pain and improve shoulder function when degenerative tendinopathy is present [76,77]. Conversely, extracorporeal shock wave therapies may be less effective for calcific rotator cuff tendinopathy, while ultrasound-guided percutaneous irrigation treatment [55,78,79,80], also known as ultrasound-guided lavage [80], seems to be more effective than physical therapies or corticosteroid injection [80].

Experiments suggest that nitric oxide, a free radical produced by different enzymes, enhances new tissue synthesis through several processes. A randomized control trial showed significant improvement in tendinopathy-induced shoulder pain and function at 12 and 24 weeks with glyceryl trinitrate (1.25 mg/24 h) compared to placebo [81]. No studies are available about the effect of the glyceryl trinitrate in greater trochanteric pain syndrome.

## 4. Arthritis

### 4.1. Definition

Arthritis is a disease of articular joints that alters the joints biochemically, structurally, and physiologically. There are many classifications for arthritis since it is a heterogeneous, degenerative disorder with multiple etiologies and presentations [82]. Primary or idiopathic arthritis is without a specific or known antecedent and primarily affects the hands, hips, knees, and spine. Secondary arthritis has an underlying cause such as acute trauma or a rheumatologic, metabolic, or infectious disease. This historical categorization has continuously been challenged by our continued understanding of this widespread disease [83]. Therefore, multiple descriptive categories have been proposed for joint size, local or generalized joint involvement, and clinical, biochemical, and radiological presentations [82]. In practice, the terms “arthritis” and “osteoarthritis” most commonly refer to the primary, degenerative “wear and tear” of chronic joint use and exclude autoimmune subtypes (e.g., psoriatic arthritis, rheumatoid arthritis, and spondyloarthritis) and acute secondary causes (e.g., gout and infection). This has remained the practical definition even though we continue to find overlaps in pathological processes [84,85].

### 4.2. Epidemiology

Osteoarthritis is a leading cause of pain and disability around the world [86]. Utilizing the 2010 Global Burden of Disease study, the WHO states that 10% of men and 20% of women over the age of 60 have symptomatic osteoarthritis [87]. The Johnston arthritis study found the lifetime risk of symptomatic knee osteoarthritis to be “nearly 1 in 2 overall” and “more than 2 in 3” for obese people [88]. Based on the United Nations’ population predictions, the WHO suspects that 130 million people will have symptomatic osteoarthritis by 2050 [89].

### 4.3. Pathophysiology

The main reason cited for the increasing prevalence and disability of osteoarthritis is our aging population. Biochemical and mechanical changes associated with aging are the greatest non-modifiable risk factors for osteoarthritis development and progression [90]. Women have a higher prevalence and severity of osteoarthritis, especially in the hands, hips, and knees. However, results suggesting that estrogen plays a role in incidence and progression have been conflicting [91]. Joint-specific variations are seen in different ethnic and racial groups, but osteoarthritis exists across all populations [92]. Multiple osteoarthritis genetic loci on genome-wide scans have been identified that may help to elucidate the pathophysiology and associated phenotypes in the future [93].

Osteoarthritis starts as a biochemical process affecting the synovium, cartilage, or subchondral bone and progresses to biochemical and anatomical abnormalities of the entire joint complex, culminating in illness [82,89,92]. The initial phases of this biochemical process consist of joint inflammation, as documented by Driban et al. through synovial fluid protein concentration analyses [94]. The joint inflammation could be initiated by mechanical trauma, metabolic changes, aging, or a combination of these factors. Obesity is thought to induce a local, mechanical trauma, especially on the knees. One unit of weight loss leads to a 4-unit reduction in knee load per step [95]. Obesity also contributes to osteoarthritis in non-weight-bearing joints of the hand, supporting the pathophysiological contribution of systemic metabolic inflammation to osteoarthritis incidence and progression [96]. Likewise, alcohol and nicotine both contribute to systemic inflammation and could potentiate pro-inflammatory mediators in the joint complex. Trauma, whether related to a major joint injury or occupational microtrauma, destabilizes and deforms the joint, worsening the mechanical forces affecting the cartilage and subchondral bone. Ligament laxity, sarcopenia (especially related to quadriceps weakness in knee osteoarthritis), and osteoporosis also contribute to the progression of osteoarthritis [1,2,92].

The synovium can be the source of multiple pro-inflammatory mediators that lead to pain and changes to the synovial fluid. The synovial fluid nourishes the avascular, aneural cartilage as well as produces hyaluronic acid (HA) and lubricin to reduce friction during movement. An increase in pro-inflammatory and catabolic products decrease the concentrations of cartilage-protecting factors and increase the production of cartilage degradation factors. There are also changes to the molecular weight of HA and a decrease in the concentration of lubricin [97].

The cartilage is also protected from mechanical stress and nourished by the subchondral bone. Early osteoarthritis bone remodeling could occur through different mechanisms: cellular signaling leading to bone remodeling and resorption and vascular invasion leading to cartilage degeneration and diminished mechanical integrity. As osteoarthritis progresses, there is a net increase in remodeled bone formation, leading to the characteristic osteophytes, sclerosis, and joint deformity seen in advanced disease [89,92].

When an imbalance exists in the structure or functioning of the extracellular matrix of the cartilage, additional inflammatory mediators and mechanical stress create a “vicious cycle” of cartilage degeneration. Over time this “low-grade” inflammation and mechanical stress limit chondrocyte production of functional collagen (collagen type I), reduce space-occupying proteoglycans, and increase inflammatory mediators. While mechanical stress can occur over time and increase with age, there is likely an inherent age-associated change in the chondrocyte phenotype that also impairs cartilage homeostasis [89,92]. This change leads to multiple molecular events and consequences (including altered gene expression related to senescence, DNA and telomere dysfunction, altered protein secretion, oxidative damage, decreased growth factor response, and apoptosis), precipitating cartilage destruction and susceptibility for osteoarthritis in the elderly [90,98].

### 4.4. Clinical Presentation

Patients with osteoarthritis can be identified through a range of common phenotypes, the presence of risk factors, clinical signs, and symptoms [99].

Patients presenting with osteoarthritis complain of short-lived (<30 min) pain and joint stiffness at the beginning of movement, especially in the morning. As osteoarthritis worsens, pain can be present after a period of activity or become continuous. Pain and stiffness create a functional limitation in movement, affecting the ability to perform activities of daily living. Primary physical exam findings can include crepitus, restricted movement, and bony enlargement (Heberden, Bouchard, and Luschka’s joint). There is no or only minimal palpable warmth, redness, or effusion in joints affected by osteoarthritis. Through the progressive increase in pain and loss of mobility, reduction in fitness and social isolation can be common [89,92]. Consistently, previous studies suggested that chronic pain and reduced fitness, common in patients with osteoarthritis, contribute to depression, obesity, cardiovascular disease, and decreased quality of life—potentiating the risk of overall morbidity and mortality [89,92].

Radiography is considered as the “gold standard” for osteoarthritis diagnosis and is commonly used to identify the severity and to monitor the progression of joint disease in symptomatic patients [89,92]. Kellgren and Lawrence [100] proposed a prominent system to classify radiographic osteoarthritis changes and look for osteophytes, joint space narrowing, subchondral sclerosis, and deformity. The absence of radiological evidence of osteoarthritis does not eliminate the possibility of disease, and the presence of radiological evidence does not directly correlate to patient symptoms. Therefore, nearly all leading osteoarthritis groups state that an appropriate diagnosis can be made on clinical presentation alone in a symptomatic elderly adult [89,92,101], though radiographs likely add diagnostic specificity [102]. Additional imaging (e.g., magnetic resonance) and laboratory findings (e.g., synovial fluid and serum analyses) rule out other causes of joint pain, assist in research advances, or prepare for surgical intervention. Furthermore, the abovementioned clinical variables, alone or in combination with radiological variables, can also be useful for patient stratification [103]. For example, a stratification based on the severity of symptoms distinguishes between asymptomatic and symptomatic arthritis sufferers, while a stratification based on imaging findings distinguishes between diffuse disorder and joint-specific disorder. Driban et al. [104] have provided recent evidence that age, glucose concentrations, body mass index, and static alignment are the most important variables for classifying individuals with incident accelerated knee arthritis. Stratification techniques based on clinical and radiological variables may be the key to developing disease-modifying interventions for subsets of patients within this heterogeneous disease [103].

### 4.5. Management

Multiple protocols for therapeutic management of osteoarthritis exist, including those from the European League Against Rheumatism (EULAR) [101], European Society for Clinical and Economic Aspects of Osteoporosis, Osteoarthritis and Musculoskeletal Diseases (ESCEO) [105,106], Osteoarthritis Research Society International (OARSI) [107], and American College of Rheumatology (ACR) [108,109]. Treatment algorithms from these organizations and others can roughly be broken into three, stepwise segments: (1) lifestyle treatments, (2) pharmacologic treatments, and (3) interventional treatments.

First, all osteoarthritis organizations support patient information and education about their condition to promote mechanical and metabolic improvements. These include self-management, weight loss, and an exercise program to strengthen the joint and supporting structures. Additionally, a psychosocial assessment is likely beneficial for patients with chronic pain. Weight loss and obesity treatment is a universal recommendation, though many recognize the difficulty of achieving success [92]. ESCEO’s comprehensive recommendations in 2014 highlighted the need for weight loss and included an endorsement of 5% weight reduction in 6 months with the goal of 10% weight loss to achieve significant symptom benefit [105]; however, ESCEO’s 2019 update admitted to a lack of evidence-based treatment regarding weight management and physical exercise [106]. Exercise programs vary in their specific recommendations and generally encourage both cardiovascular and strength training (specifically quadriceps strengthening) [110]. Physical therapy and supervised, progressive exercise programs are encouraged, especially if they successfully transition into a self-administered home program [111]. Aquatic exercises are useful, especially if land-based activities are too painful [108]. A walking cane can help with functional movement when pain is present [106,108]. Braces, splints, and taping can be used for comfort or to provide mechanical support of a deformity depending on the location of the offending joint [110]. Recommendations for wedged insoles are mixed, but generally negative due to limited improvement and possible side effects [110]. Guidelines are also mixed regarding the most common physical therapy modalities, including thermal (heat/cold) application, manual therapy, acupuncture, and electrotherapies [112].

Pharmacologic treatment recommendations vary depending on the joints affected and patient comorbidities. The most common initial treatment recommendation is acetaminophen (paracetamol) due to its favorable side-effect profile [110]. ESCEO diverges from this recommendation (due to paracetamol’s minimal benefit and possible side effects) and recommends the use of symptomatic slow-acting drugs for osteoarthritis (SYSADOAs) with prescription grade-only crystalline glucosamine sulfate or chondroitin sulfate as a first-line pharmacologic treatment of symptomatic osteoarthritis [106]. This was not supported by neither the 2012 nor the updated 2019 ACR recommendations [108,109], but it should be noted that prescription-grade SYSADOAs are not available in the United States. Capsaicin is a topical agent with mixed support that could be used if tolerated [107]. If the patient is still symptomatic, NSAIDs are considered by all osteoarthritis treatment algorithms [110]. Due to bleeding, renal, and cardiovascular risks, topical NSAIDs should be considered first for superficially located joints [106,107,110]. COX-2 inhibitors with a proton-pump inhibitor should be considered in the setting of gastrointestinal risk. However, COX-2 inhibitors should be avoided in patients with known cardiovascular risks. Patients with glomerular filtration rate < 30 cc/min should avoid all NSAIDs [106]. Opioids, especially tramadol, are recommended for severe pain, but should be used with caution due to concerns of addiction, abuse, and diversion [106,107,108,109]. OARSI, ESCEO, and ACR additionally support duloxetine for patients with multiple symptomatic joints and signs of central sensitization [106,107,108,109].

Interventions for osteoarthritis typically begin with intraarticular injections and can escalate to total joint replacement. Corticosteroid injections can temporarily help with pain (<3 weeks) and are generally recommended when there are additional signs of inflammation like breakthrough pain or a mild effusion, but not on a continuous basis [106,107,109,110]. Intra-articular hyaluronate (viscosupplementation) can also improve pain and joint inflammation and is a popular alternative to steroids. Formal recommendations, however, remain controversial due to mixed research results, cost, and availability; the most recent guidelines (by ESCEO and ACR) offer no to low-strength recommendations for its use in the hip and knee [106,109,110]. With our aging population, total joint replacements are becoming more prevalent [113]. Surgical intervention remains reserved for patients with daily pain despite conservative treatment to ensure the benefits outweigh the risks [89,92].

## 5. Conclusions

We reviewed the literature relative to three inter-related musculoskeletal disorders (i.e., sarcopenia, tendinopathies, and arthritis) that are highly prevalent in elderly subjects, in order to highlight the pathophysiological common denominator and propose strategies for personalized clinical management of patients presenting with this combination of disorders. Even though their clinical presentation can be different (loss of skeletal muscle mass and function in sarcopenia, persistent tendon pain and loss of function in tendinopathies, and persistent joint pain and stiffness in arthritis) [7,36,89,92], the three disorders have common pathophysiological and clinical characteristics. On the basis of the general content of this article, we report in the following a non-comprehensive list of highlights relative to the common pathophysiological and clinical characteristics of the three disorders.

1. The progressive loss of (focal or generalized) neuromuscular performance is the pathophysiological denominator common to the three disorders [7,13,22,24].
2. The three disorders increase the risk of adverse outcomes such as pain, motor impairment, increased risk of falls and fractures, impaired ability or disability to perform activities of daily living, and reduced quality of life [13,59,89,92].
3. The three disorders have a heterogeneous clinical presentation: patient stratification based on clinical and imaging variables may be the key to developing disease-modifying interventions for different subsets of patients [103,104].
4. The precise management of the three disorders requires not only the use of available tools and recently proposed operational definitions, but also the development of new tools and approaches for prediction, diagnosis, monitoring, and prognosis of the three disorders and their combination.
5. Physical exercise, alone or in combination with nutritional interventions, is the approach currently recommended as the primary treatment of the three disorders [24,25,49,67,107].
